# Sustainable Solution for Plastic Pollution: Upcycling Waste Polypropylene Masks for Effective Oil-Spill Management

**DOI:** 10.3390/ijms241512368

**Published:** 2023-08-02

**Authors:** Junaid Saleem, Zubair Khalid Baig Moghal, Rana Abdul Shakoor, Gordon McKay

**Affiliations:** 1 Division of Sustainable Development, College of Science and Engineering, Hamad Bin Khalifa University, Qatar Foundation, Doha P.O. Box 34110, Qatar; gmckay@hbku.edu.qa; 2 Center for Advanced Materials, Qatar University, Doha P.O. Box 2713, Qatar; zubairkhalid009@gmail.com (Z.K.B.M.); shakoor@qu.edu.qa (R.A.S.)

**Keywords:** microporous, polypropylene, plastic waste, microplastic, pouch, oil sorbent, face masks

## Abstract

The use of Polypropylene PP in disposable items such as face masks, gloves, and personal protective equipment has increased exponentially during and after the COVID-19 pandemic, contributing significantly to microplastics and nanoplastics in the environment. Upcycling of waste PP provides a useful alternative to traditional thermal and mechanical recycling techniques. It transforms waste PP into useful products, minimizing its impact on the environment. Herein, we synthesized an oil-sorbent pouch using waste PP, which comprises superposed microporous and fibrous thin films of PP using spin coating. The pouch exhibited super-fast uptake kinetics and reached its saturation in fewer than five minutes with a high oil uptake value of 85 g/g. Moreover, it displayed high reusability and was found to be effective in absorbing oil up to seven times when mechanically squeezed between each cycle, demonstrating robust oil-sorption capabilities. This approach offers a potential solution for managing plastic waste while promoting a circular economy.

## 1. Introduction

Plastics, due to their low cost, easy formability, hydrophobicity, light weight, and durability, are rapidly used in medical equipment, electronic devices, packaging, and many other fields [1,2,3]. Over the past 60 years, plastic production has increased from 2-million tons to 360-million tons, according to reports [4,5]. As a result of high collection costs and a lack of infrastructure, approximately 50% of plastic becomes waste after a single use [6]. Despite the convenience of plastic products, there has been a growing concern about the environmental challenges resulting from white pollution. According to estimates, there will be 12-billion tons of plastic waste in landfills and the natural environment by 2050 [7]. Generally, plastic waste is composed of various polymers, such as polystyrene (PS), polypropylene (PP), polyethylene (PE), polyethylene terephthalate (PET), and polyvinyl chloride (PVC) [8].

In the aftermath of the COVID-19 pandemic, plastic waste, particularly PP waste, has increased significantly. A significant part of this rise is due to the use of single-use plastic items, especially personal protective equipment (PPE) such as masks, gloves, and isolation gowns [9,10,11]. Many countries have implemented measures [12,13,14,15] to reduce exposure to the COVID-19 virus and ensure the safety of their healthcare workers as well as citizens, including mandatory mask-wearing and an increased use of disposable items [16,17]. According to estimates, the global production of plastic waste has increased since the beginning of the pandemic, with an estimated daily output of 1.6-million tons. Additionally, it was reported that Asian countries alone used approximately 289.63-billion single-use plastic (SUP) facemasks annually, while European countries used 61.02-billion SUP facemasks as a measure to prevent the transmission of viruses [18,19,20,21]. This has resulted in a surge in plastic waste, which has placed a significant burden on waste management systems and the environment. In particular, it was reported that face masks are a source of microplastics and nanoplastics [10,22,23]. In summary, a total of 8.4-million tons of plastic waste related to the pandemic have been produced across 193 countries as of August 2021. Out of this, approximately 25,900 tons have been released into the world’s oceans, comprising roughly 1.5% of the overall plastic waste discharged from rivers globally [24].

PP is a commodity thermoplastic [25] used in the production of various single-use supplies, including syringes and other medical devices [26,27], masks, gloves, and other protective gear [28,29]. A study showed that PP waste generated from a single surgical mask and N95 mask is around 4.5 g and 11 g, respectively [19]. Additionally, the most widely used real-time polymerase chain reaction (RT-PCR) confirmatory test for COVID-19 generates approximately 37 g of plastic residue, of which 89.99% corresponds to PP per tested sample [30]. Prior to COVID-19, PP was still one of the most widely used plastics with a global production rate of 55 × 10^6^ tons a year, of which only 1% is recycled [31,32]. Unfortunately, PP is non-biodegradable and can take hundreds of years to decompose, making it a significant environmental threat [33]. Landfilling sites can cause soil and water contamination and have limited capacity, and the increasing amount of PP medical waste generated during the COVID-19 pandemic has put additional strain on already overloaded disposal sites [34,35]. Moreover, improper disposal of these items can lead to plastic pollution in oceans and other waterways, causing harm to marine life and ecosystems [36].

In recent years, several recycling methods such as mechanical, chemical, and thermo-chemical recycling have been proposed to reutilize and mitigate PP medical waste [37,38]. Although mechanical recycling using extrusion is found to be an effective method of reducing plastic waste and conserving resources, the process often degrades the polymer properties [39,40]. In several studies, additives have been suggested for composites made from disposable masks, such as multi-walled carbon nanotubes for conductive polymers [41], luffa sponge powder 3D-printing feedstock, low-value building materials with enhanced mechanical properties [42], and natural fibres for food packaging, which possess good microbial resistance with improved tensile and elongation properties [43]. However, medical waste plastic is challenging to recycle due to contamination, which can cause health hazards and a further reduction in the quality of the recycled material, and needs pre-treatment to avoid these issues [44,45,46].

In contrast, chemical recycling of polyolefins involves breaking C-C bonds to convert the polymer back into its constituent monomers, such as ethylene or propylene, for which a massive amount of heat energy is required due to the endothermic nature of the reaction. In addition, the process itself and contamination may lead to the emission of undesired greenhouse gases. However, the thermo–chemical process is used for the recovery of heat energy [47,48,49], as in the case of incineration, or to produce oil and gas [50,51], as in the case of pyrolysis. As a result, it is crucial to upcycle PP waste and transform it into new value-added materials with improved properties for specific use [52].

Recent research suggested the conversion of PP-based disposable facemasks into hard low-density carbon material to be used as anode materials for sodium-ion batteries through sulfonation and subsequent carbonization at high temperatures, up to 2400 °C [53]. Another similar study showed the conversion of disposable medical masks into porous carbon with a high specific surface area for use in electrochemical applications such as batteries and super capacitors [54,55]. Moreover, researchers have developed activated carbon material with a highly porous structure for the adsorption of CO_2_ [56]. Contrary to energy-consuming methods, one study suggested a technique in upcycling PP waste via the fabrication of an engineering product with improved thermo–mechanical properties by blending non-woven PP fibre from facemasks, with acrylonitrile butadiene rubber and maleic anhydride as the compatibilizers. This would result in an improved interfacial adhesion [57]. Another sustainable research upcycled the used biomedical disposable facemasks into high-value nano-filtration membrane materials with the dissolution–precipitation method. The resulting membranes exhibited good stability and filtration performance when tested on roxithromycin (an active pharmaceutical ingredient) and rose bengal (a dye) [58]. A further application of upcycling PP facemask waste that needs attention is the synthesis of oil sorbents for wastewater treatment, considering PP’s hydrophobic properties.

Water pollution is increasing tremendously due to oil spills on the water surface and shoreline. Using oil sorbents is one way to combat these spills. Effective sorbents have high selectivity, super-fast uptake kinetics, high retention rates and oil uptake, and enough mechanical strength to enable effective application. In this context, polymers, carbon nanotubes, and graphene have been widely explored as synthetic sorbents in the form of aerogels, thin films, pads, and non-woven fibres. Polymers such as polyurethane (PU) have been used as an oil sorbent with an uptake capacity of 100 g/g and a residual sorption capacity of 70% after 15 consecutive sorption-squeezing cycles [59]. However, the disadvantage of PU is that it is a specialty polymer; it is extremely expensive compared to PP [60]. Secondly, PU constitutes less than 3% of total plastic waste compared to PP, which is 20% [61,62]. Consequently, low-cost, hydrophobicity, and high plastic waste content make PP a natural choice as an oil-sorbent material. Moreover, aerogels based on polymers such as PP, polyvinylidene fluoride (PVDF), polycarbonate (PC), high-density polyethylene (HDPE), and PU were also reported with limited oil uptake capacity ranges from 6 to 25 g/g [63,64,65,66,67].

Recent research has shown that graphene aerogels exhibit remarkable oil uptake capabilities, but their practical application is hindered by their low mechanical strength and inability to be recycled through simple mechanical squeezing. They require chemical treatment to extract the oil that is trapped inside the 3D porous structure, which further adds to their impracticality [63,68,69]. Moreover, the current methods of converting graphite to graphene significantly increase the overall cost of production. In contrast, carbon nanotubes have also been used to utilize high surface area, but the cost of production is extremely high for commercial applications [63,70]. The reported methods require a long-winded preparation process and rely on expensive nanoparticles as raw materials.

In searching for the solution to oil-spill problems using plastic waste generated during the COVID-19 pandemic, one study suggests the surface modification of PP discarded facemasks by alkane solvents to enhance the hydrophobicity for oil adsorption. This study proposed the use of KF94 masks, which have the highest PP content. When these PP face masks were treated with heptane at 90 °C for 1 hr, they exhibited a maximum oil-removal capacity of up to 21 g/g from the water surfaces [71]. A more complex method was developed later on utilizing multiple organic solvents and by deposition of a superhydrophobic fluorine-free metal–azolate framework (MAF-6) on PP fabric extracted from disposable face masks for cleaning up the oil spill. It was determined that the adsorption capacity for unbranched and cyclic alkanes reached 14 g/g, but the affinity for low-weight alkanes was much lower [72].

To address these issues, upcycling PP waste of all types of single-use face masks to oil-sorbent pouches with a less complex process and enhanced sorption capacity has been proposed in this work as a potential solution, in addition to waste-to-waste reduction. The oil sorbent comprises superposed fibrous and microporous thin films sealed in a pouch made from a fibrous thin film. The pouch is capable of selectively sorbing oils in the presence of water, resulting in excellent sorption performance. Furthermore, the sorbent can be reused multiple times without the need for chemical treatments or complex methods. Compared to other synthetic sorbent materials, this oil sorbent pouch made from upcycled PP waste is low-cost, abundant, and readily available. In addition to prior processing, the collected plastic waste was isolated and quarantined for 14 days because most pathogens, especially viruses, are dependent on host cells for their nutrients to survive and reproduce in specific environmental conditions, such as temperature, pH, and moisture levels [73,74,75]. Additionally, the pathogen cannot thrive effectively on surfaces like plastics; isolating it for an effective time would be worthy to mitigate the pathogens before processing [76]. Furthermore, the process involved the dissolution of PP waste in an organic solvent at a temperature of ~130 °C, inhibiting the spread of the viruses [75,77]. The environmental impacts of preparing pellets by dissolving waste PP in Xylene at 130 °C is lesser than the virgin PP pellets prepared from the petroleum source [78].

## 2. Materials and Methods

### 2.1. Materials and Characterizations

Waste PP masks were collected from the local site. An isomeric mixture of xylene (CAS Number: 1330-20-7) was purchased from VWR Chemicals and used as received. The glass substrate plates were cut to a size of 5 cm × 4 cm and can be fixed on the spin coater’s chuck. PP non-woven sheet was bought from a local vendor. Heating and annealing were performed in a hot-air oven (MINO/30/TDIG) by Genlab Ltd (Tanhouse La, Widnes, UK). Hot plate annealing was carried on Heidolph magnetic hot plate stirrer.

FEI Quanta650FEG was used to capture scanning electron microscope (SEM) images. PANalytical Empyrean multipurpose XRD was used to measure the XRD studies. Thermo Fischer Escalab 250XI platform(Thermo Fisher Scientific, Waltham, MA, USA) was used to measure X-ray photoelectron spectroscopy (XPS). A monochromated X-ray source (Al Kα: 1486.6 eV) was used. FTIR was conducted using the PerkinElmer Frontier instrument (PerkinElmer, Waltham, MA, USA). Profilometry imaging was conducted using a Leica DCM8 microscope (Leica Microsystems Ltd, Wetzlar, Germany). The optical contact angle was calculated using OCA 35, Dataphysics Instruments GmbH–Filderstadt, Germany. A micrometer was used to measure the thickness, and it was cross-checked with Deflesko FS3 PosiTector 6000 (New York, NY, USA) having a ferrous metal base. Oxford Instruments’ Goleta, CA, USA AFM were used to measure the data.

### 2.2. Synthesis of Sorbent

PP plastic waste was obtained and washed with detergent and water to remove any dirt or oil. PP plastic waste was isolated and quarantined for 14 days before processing to mitigate pathogen risk. 1.2 g of PP was then dissolved in 10 ml of xylene at 130 °C for 20 min, or until a clear solution was obtained. Simultaneously, a glass plate was heated to 120 °C and placed on a spin-coater chuck. The hot solution was poured on a hot glass plate and allowed to spin. The spin coating was programmed in three steps to achieve a uniform thin film. (a) Ten seconds of rotation at 400 rpm are included; (b) For 60 s, a rotational speed of 1000 rpm was maintained; and (c) Rotational speed was kept constant at 3000 rpm for the next 120 s. To collect excess solution and polymer, they were drained. The glass substrate with the thin film was detached from the chuck and in a hot air oven. It was then subjected to annealing for 20 min at 160 °C. Thus, it resulted in PP porous-thin films. To prevent solvent exposure to the environment, the stirrer and spin coater are placed side-by-side inside the fume hood. Three stages were programmed for the spin coating. Due to the ease of binding the polymer to the glass substrate, the first stage was conducted at a low speed, followed by a medium speed. This step facilitates uniform thickness distribution by spreading the polymer. As a final step, the speed was further increased to remove the solvent molecules and refine the thickness of the film.

**Figure 1:**
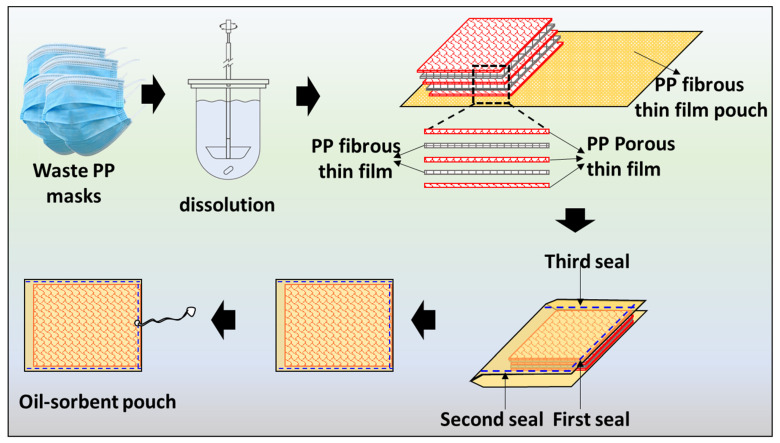
Preparation of a waste PP-based oil-sorbent pouch.

The PP fibrous-thin film was made by heating a PP non-woven sheet in a hot-air oven at 80 °C for 5 min to enhance the flexibility and compatibility with the PP porous-thin film. It was then sandwiched between a PP porous-thin film. These PP fibrous-thin film sheets act as a support and facilitator for oil penetration and seeping. The superposed films were placed in a pouch made from PP fibrous-thin film. The pouch facilitated easy handling in oil sorption. The pouch was heat-sealed in three fronts, as shown in Figure 1. Of these three fronts, the side of the pouch with the hoop is sealed in such a way that one edge of the PP porous-thin film with a facilitator is placed in between, which immobilizes the film, enabling it to firmly sorb oil without the risk of tearing. Moreover, for ease of use, a thread was knitted to one end of the pouch.

The oil-sorbent pouch contained five layers. The first, third, and fifth layers were PP porous-thin films, and the second and fourth were PP fibrous-thin films. These were then placed inside the pouch and sealed. Each film had a dimension of 20 cm^2^, and the pouch was made slightly bigger to accommodate these films. Packing and sealing were conducted to prevent damage of the films during sorption and desorption studies.

### 2.3. Porosity Measurement

The porosity was calculated by the volume of the solvent, i.e., N-Methyl-2-pyrrolidone penetrated into the porous structure of the sorbent. Initially, the weight of the sorbent was noted, and then the sorbent was placed in the solvent until it reached saturation. Then, the sorbent was wiped with tissue to remove surficial solvent, and the wet sorbent was weighed. The difference between the wet sorbent and dry sorbent determines the volume of the solvent sorbed. The densities of the solvent and sorbent were adjusted with the volume to determine the porosity. Ethanol can also be used as a solvent. This method gives an approximation, which is sufficient in our study.

### 2.4. Contact Angle Measurement

For this study, 2–6-μL waterdrop was utilized for contact angle measurements. All reported values were the average from five measurements.

## 3. Results

### 3.1. SEM and AFM Analysis

**Figure 2:**
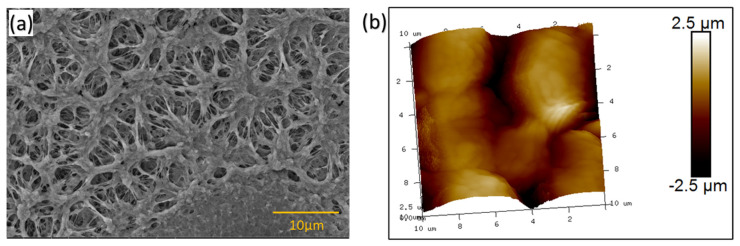
PP porous-thin film (**a**) SEM image; (**b**) AFM image.

The surface morphology and structure of a prepared thin film was evaluated using SEM analysis. In particular, it was used to study the pore size, structural distribution, arrangement, and pore shape of the thin film to better understand its physical characteristics. The thin film had a porous structure with a porosity of approximately 40%. The pores in the thin film were observed to be elliptical in shape with a size range of 0.5 µm to 5 µm, as shown in Figure 2a. Additionally, these pores were found to be connected through intermolecular interactions of polymer chains, forming a network within the thin film, as explained elsewhere [79].

As shown in Figure 2b, the surface roughness of the PP sorbent was evaluated using AFM. An AFM analysis revealed that the surface roughness (RMS) of the PP porous-thin film was 749 nm, which corresponds to the high roughness of the surface that facilitates oil sorption.

### 3.2. Microscopy Images

**Figure 3:**
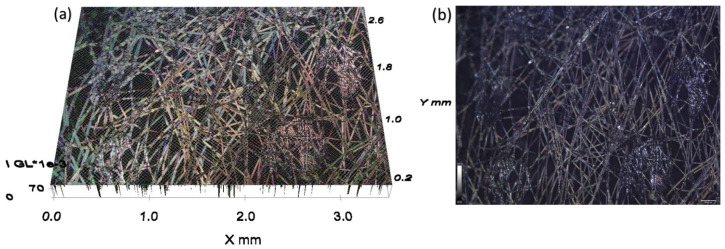
Bright-field optical microscopic imaging of PP-fibrous thin film. (**a**) Tilted angle; (**b**) Top view.

The microscopy images of the PP fibrous-thin film were captured using a bright field imaging under a microscope, which showed the non-woven pattern of the PP fibrous-thin film (Figure 3a,b). Its highly porous network facilitated faster oil penetration. Thus, this material was used in the making of a pouch that carries thin films and is the facilitator for easy penetration.

### 3.3. XPS Analysis

**Figure 4:**
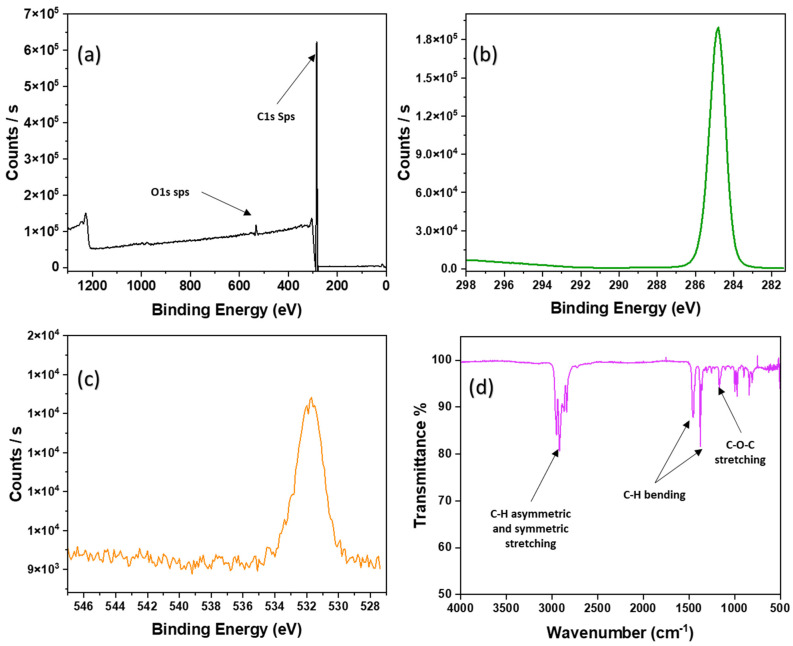
PP porous-thin film after heat. (**a**) XPS survey spectra; (**b**) High-resolution XPS C1s spectra; (**c**) High-resolution XPS O1s spectra; (**d**) FT-IR spectrum.

PP porous-thin film high-resolution XPS survey spectra, Carbon 1s, and Oxygen 1s spectra are shown in Figure 4a–c. To investigate the chemical composition of the polymeric surfaces and to determine the presence of solvent molecules in thin films, XPS analysis was conducted. The peaks at 285 eV and 532 eV correspond to carbon species and oxygen species, respectively. Figure 4b,c show XPS spectra of C1s and O1s at high resolution. As mentioned earlier, to synthesize PP porous-thin films, Xylene (a non-polar solvent with an aromatic character) was used as a solvent to dissolve PP. A binding energy peak at 292 eV indicates the existence of xylene. It is usually seen that the residual solvent exhibits π–π* interactions of C=C of the aromatic peak and is strongly observed at 292 eV [80]. The absence of a peak at 292 eV refers to the complete removal of the solvent xylene that is used in our methodology. However, the thin film may have partially oxidized during the heating process, as indicated by the existence of a minor peak of oxygen at ~532 eV. Yet the oxygen peak also facilitates intermolecular interactions. However, the oxygen content is less than 2%, hence it does not alter the overall hydrophobicity of the PP porous-thin film.

### 3.4. FT-IR Analysis

We further confirmed the results obtained from XPS and SEM analysis using FT-IR analysis. The peaks at 1456 cm^−1^ and 1376 cm^−1^ in the FT-IR spectrum indicate the presence of C-H bending, and the peak at 728 cm^−1^ determines the C-H rocking peak of the PP. The four peaks in the region 2950–2850 cm^−1^ were attributed to the C-H asymmetric and symmetric peaks, as well as the stretching bands of branched methyl groups. However, the peak at 1235 cm^−1^ indicates the presence of C-O stretching bonds of ether linkage that interconnect the polymer chains through intermolecular interactions (Figure 4d).

### 3.5. DSC and XRD Analysis

**Figure 5:**
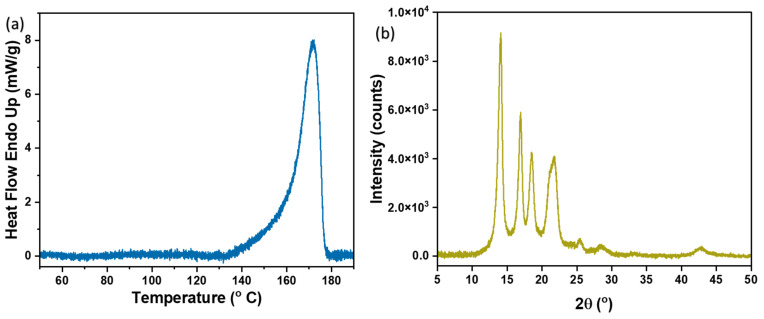
PP porous-thin film analysis. (**a**) DSC and (**b**) XRD.

Additionally, to evaluate the change in the enthalpy and crystallinity of the pure PP crystal and thin film, DSC studies were performed. Pure isotactic PP crystals and the as-prepared thin film exhibit enthalpy changes of 207 J/g [81] and 118 J/g, respectively. The enthalpy change of 118 J/g for the thin film corresponds to the semi-crystalline structure of PP, having a crystallinity of 57% [82]. Meanwhile, DSC was also used to determine the melting point of the PP porous-thin film, which was found to be 168 °C (Figure 5a).

Furthermore, XRD analysis is also used to reveal the crystallinity of the PP porous-thin film. This can be seen in the form of characteristic peaks at 14°, 16.9°, 18.4°, and 21.7° of PP [83]. However, the crystallinity from XRD has been determined to be 60%, which corresponds to the crystallinity determined by the DSC analysis (Figure 5b).

### 3.6. Contact Angle

**Figure 6:**
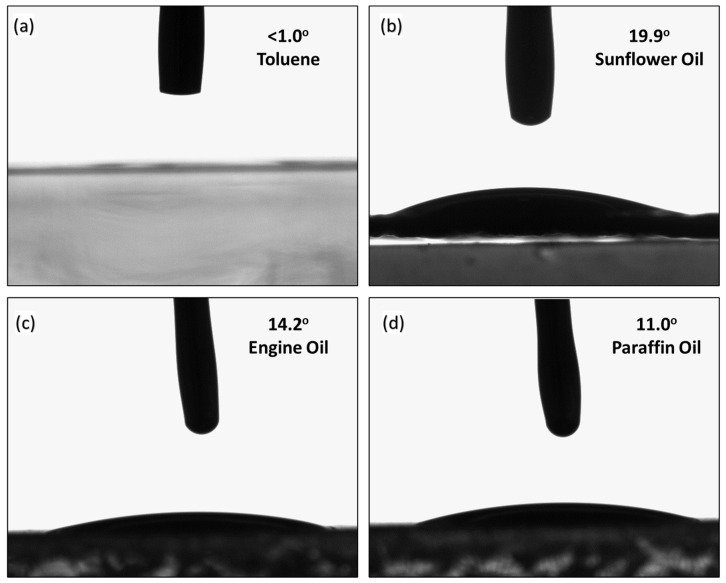
Contact angle analysis for (**a**) Toluene; (**b**) Sunflower oil; (**c**) Engine oil; and (**d**) Paraffin oil. The size of the drop was 5 µL.

To evaluate the hydrophobicity, hydrophilicity, or oleophilic nature of the waste PP-based oil-sorbent pouch, the contact angle measurements were conducted with different oils. In this context, four different oils are used to calculate the contact angle of the oil-sorbent pouch, which includes toluene, sunflower oil, engine oil, and paraffin oil. When tested with toluene, the contact angle was found to be <1°. Hence, it was difficult to capture the contact angle. As soon as the droplet comes in contact with the oil-sorbent pouch surface, the toluene spreads over the thin film in a fraction of a second without creating any meniscus. This flattening suggested that these films can absorb organic solvents. However, the contact angle for sunflower oil, engine oil, and paraffin oil was found to be 19.9° ± 2.3°, 14.2° ± 1.3°, and 11° ± 2.6°, respectively. We could not conduct the contact angle of the crude oil because of the viscous and sticky nature, resulting in the blockage of the needle. However, we assume it to be below 30° and superoleophilic. The results obtained from the contact angle analysis demonstrate the superoleophilic nature of the suggested oil-sorbent pouch towards oil samples (Figure 6a–d and Table 1).

### 3.7. Comparison of Oil-Sorption Capacity Using Thin Films with and without Pouch

#### 3.7.1. Oil Saturation

**Figure 7:**
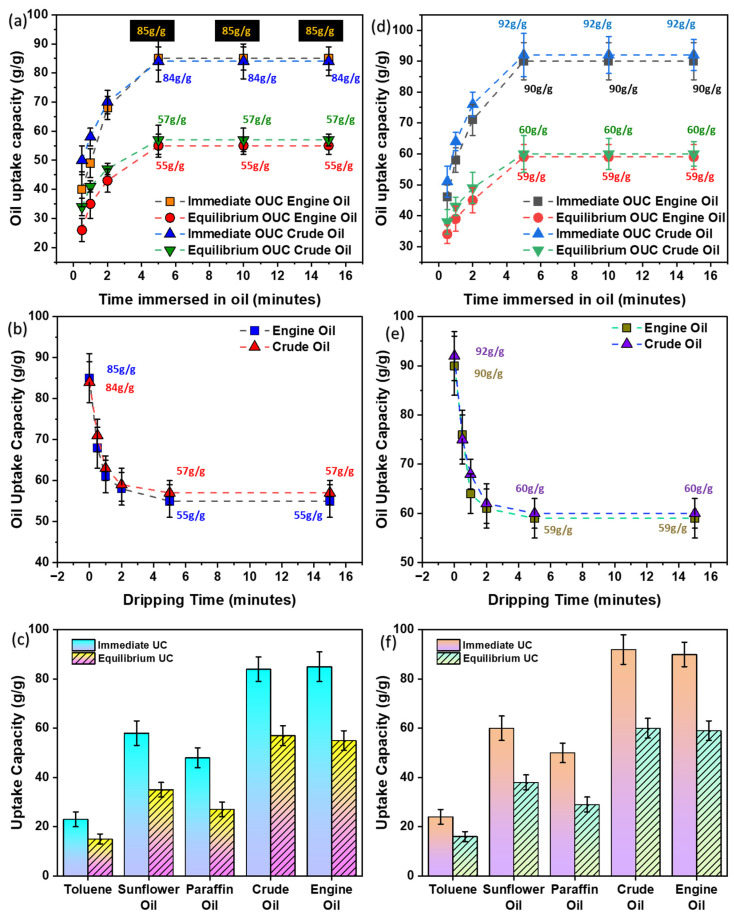
(**a**–**c**) Oil-sorption capacities corresponding to waste PP-based oil-sorbent pouch. (**a**) Saturation time using engine oil; (**b**) Dripping time using engine oil; (**c**) Comparison of uptake capacities using different oils; (**d**–**f**) Oil-sorption capacities corresponding to waste PP-based oil-sorbent without pouch. (**d**) Saturation time using engine oil; (**e**) Dripping time using engine oil; (**f**) Comparison of uptake capacities using different oils, (OUC = oil uptake capacity; UC = uptake capacity).

Essentially, saturation kinetics refers to the time used by the oil-sorbent pouch to reach a saturation point, where the oil can no longer be absorbed. Saturation kinetics revealed that the as-prepared oil sorbent reached its saturation within 5 min of contact with oil. The saturation capacity at different times is shown in Table 2 and Figure 7a. Saturation kinetics was conducted by immersing the sorbent in the oil for a specific time and removing it. The weight of the sorbent was then measured. The difference between the initial weight and the absorbed weight provides the uptake capacity.

#### 3.7.2. Oil Dripping

Dripping kinetics is the amount of oil dripped from the waste PP-based oil-sorbent pouch to time. When an oil-sorbent pouch is placed or dipped in oil, it absorbs oil and allows it until it reaches saturation. After reaching equilibrium, the oil sorbent is removed, and loosely connected oil is allowed to drain. Initially, the oil drips more than usual, but as time passes, the dripping decreases. Eventually, some oil is retained by the film, and the oil does not drip anymore. This is termed as retention capacity of the oil-sorbent pouch. The as-prepared oil sorbent reached its equilibrium uptake value within 5 min of dripping, i.e., after 5 min, there will be no more dripping of oil from the sorbent. The retention capacity or dripping kinetics at different times are presented in Table 3 and Figure 7b.

### 3.8. Comparison with Different Oils

The uptake capacity of the prepared waste PP-based oil-sorbent pouch was investigated using different oils and liquids, which include toluene, sunflower, paraffin, crude, and engine oils. These oils were evaluated in the context of their uptake capacity at an immediate time and equilibrium. The uptake capacity was measured soon after the saturation is achieved and also allowed to drip for 5 min after reaching equilibrium. The results have demonstrated the maximum oil uptake capacity for engine oil, with an 85 ± 6 g/g oil uptake for immediate withdrawal and 55 ± 4 g/g oil uptake at equilibrium. Amongst all, the engine oil showed the maximum sorption, hence it was used for further studies. The oil uptake capacity calculated “immediately” and at “equilibrium” are presented in Table 4 and Figure 7c.

Furthermore, the oil-sorption capacity of the synthesized superposed thin films (without a pouch) was also evaluated. Consequently, the superposed thin films showed an increased oil-sorption capacity due to their lower initial weight. The results are shown in Figure 7d–f.

### 3.9. Oil–Water Separation

**Figure 8:**
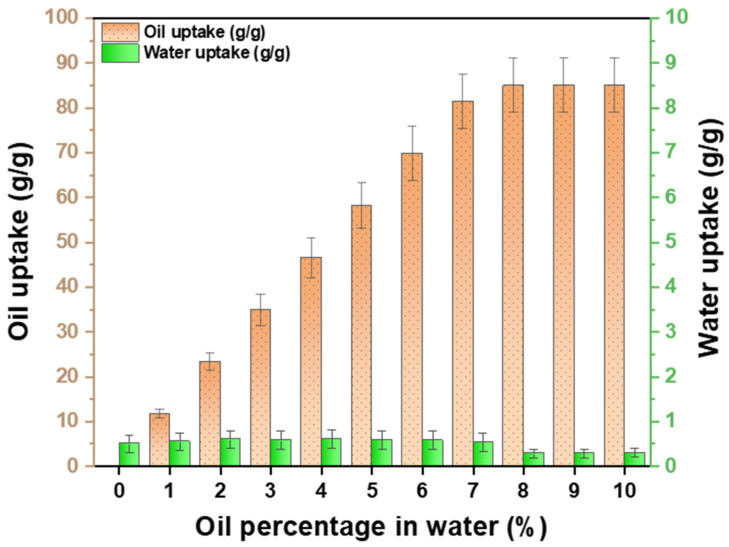
Bar graphs showing the selectivity of the prepared oil sorbent towards oil in the presence of different percentages of oil in water.

The oil sorbent was evaluated for selectivity in the presence of water. Different concentrations of engine oil in water ranging from 1% (100 mg/10 mL) to 10% (1000 mg/10 mL) were studied. The efficiency of the oil-sorbent pouch was evaluated by placing it on an oil film spread over the water surface. The oil-sorbent pouch was evaluated for its oil-in-water sorption capacity in various oil–water concentrations. The results showed that a fixed amount of 0.5 g/g of water was absorbed by the pouch in all the studied oil–water concentrations. When the oil–water concentrations exceeded 8%, the oil-sorbent pouch solely absorbed oil. Conversely, in cases where the oil–water concentrations were less than 8%, the pouch absorbed two-to-three droplets of water, which could be readily displaced by oil. The results are reported in Table 5. The film had a 99.5% oil–water separation efficiency (Figure 8).

### 3.10. Recyclability

**Figure 9:**
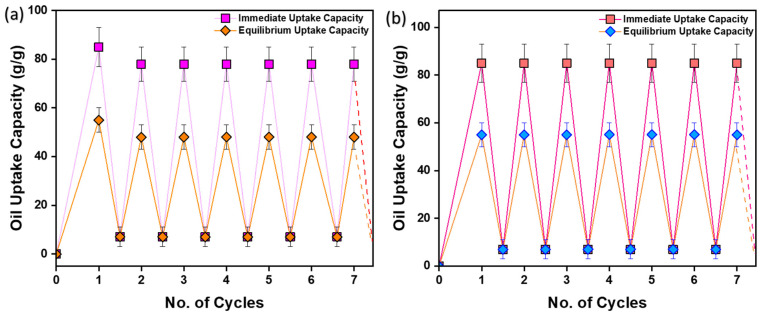
Recyclability study of the prepared oil-sorbent pouch using; (**a**) Mechanical squeezing; (**b**) Chemical squeezing.

The recyclability of the pouch using mechanical squeezing and hexane washing is presented in Figure 9. A sample with an area of 20 cm^2^ was used. The sorbent pouch was dipped in engine oil until saturation was achieved. The oil-sorbent pouch was then removed from the oil and weighed immediately. The oil uptake capacity measured immediately after taking out the pouch from the oil surface was found to be 85 g/g, whereas the oil-uptake capacity after dripping for 5 min until equilibrium was found to be 55 g/g. Then, the sorbent pouch was mechanically squeezed by pressing the upper and lower surface by hand to remove oil and weighed again. Mechanical squeezing results in 91% oil recovery as some oil (8 g/g) remains inside the pores of the sorbent film, whereas hexane washing leads to 100% oil recovery. The sorbent film can be reused as many times as possible, with 100% and 91% efficiency using hexane washing and mechanical squeezing, respectively.

## 4. Conclusions

We synthesized an oil-sorbent pouch using waste PP, (non-woven face masks in particular, which demonstrated excellent oil-sorption capabilities). The material’s hydrophobic and oleophilic properties facilitated oil sorption, resulting in an oil uptake of 85 g/g. It exhibited elliptical-shaped pores ranging in size from 0.5 µm to 5 µm. The pouch’s high reusability, with the ability to be reused up to seven times, further enhances its potential as a sustainable solution for managing plastic waste and promoting a circular economy. These findings provide insights into developing effective methods for managing plastic waste and reducing the amount of plastic waste by upcycling.

## Figures and Tables

**Table 1:** Contact angle of waste PP-based oil-sorbent pouch with various fluids.

Fluid	Contact Angle (°)
Toluene	0.7 ± 0.2
Sunflower oil	19.9 ± 2.3
Engine oil	14.2 ± 1.3
Paraffin oil	11.0 ± 2.6

**Table 2:** Saturation capacity profile of waste PP-based oil-sorbent pouch using engine oil and crude oil.

S.No.	Saturation Time (min)	Immediate Oil Uptake Capacity (g/g)		Equilibrium * Oil Uptake Capacity (g/g)	
		Engine Oil	Crude Oil	Engine Oil	Crude Oil
1	0.5	40 ± 6	50 ± 5	26 ± 4	34 ± 3
2	1	49 ± 5	58 ± 3	35 ± 5	41 ± 2
3	2	68 ± 4	70 ± 4	43 ± 4	47 ± 2
4	5	85 ± 4	84 ± 7	55 ± 4	57 ± 5
5	10	85 ± 4	84 ± 6	55 ± 3	57 ± 4
6	15	85 ± 4	84 ± 4	55 ± 3	57 ± 2

* Equilibrium is measured after dripping for 5 min.

**Table 3:** Retention capacity of waste PP-based oil-sorbent pouch using engine oil and crude oil.

S.No.	Dripping Time (min)	Oil Uptake Capacity (g/g)	
		Engine Oil	Crude Oil
1	0	85 ± 6	84 ± 5
2	0.5	68 ± 5	71 ± 3
3	1	61 ± 4	63 ± 3
4	2	58 ± 4	59 ± 4
5	5	55 ± 4	57 ± 3
6	15	55 ± 4	57 ± 1

**Table 4:** Uptake capacity with different oils and liquids.

Oil	Immediate Uptake Capacity (g/g)	Equilibrium * Uptake Capacity (g/g)
Toluene	23 ± 3	15 ± 2
Sunflower Oil	58 ± 5	35 ± 3
Paraffin Oil	48 ± 4	27 ± 3
Crude Oil	84 ± 5	57 ± 4
Engine Oil	85 ± 6	55 ± 4

* Equilibrium is reached after draining for 5 min.

**Table 5:** Oil–water separation efficiency.

S. No.	Oil in Water (%)	Efficiency%
1	1	99.5 ± 0.3
2	2	99.5 ± 0.4
3	3	99.5 ± 0.4
4	4	99.5 ± 0.5
5	5	99.5 ± 0.4
6	6	99.5 ± 0.3
7	7	99.6 ± 0.4
8	8	100 ± 0.3
9	9	100 ± 0.2
10	10	100 ± 0.2

## Abbreviations

PP	Polypropylene
COVID-19	Coronavirus Disease of 2019
PS	Polystyrene
PE	Polyethylene
PET	Polyethylene terephthalate
PVC	Polyvinyl chloride
PPE	Personal-protective equipment
SUP	Single-use plastic
RT-PCR	Real-time polymerase chain reaction
CO_2_	Carbon dioxide
PU	Polyurethane
PVDF	
polyvinylidene fluoride	
PC	Polycarbonate
HDPE	High-density polyethylene
MAF-6	Metal–azolate framework
SEM	Scanning electron microscopy
AFM	Atomic force microscopy
RMS	Root mean square surface roughness
XPS	X-ray photoelectron spectroscopy
C1s	Carbon 1 species
O1s	Oxygen 1 species
FT-IR	Fourier transform infrared
DSC	Differential scanning calorimetry
XRD	X-ray diffraction
S.No.	Serial number
UC	Uptake capacity
OUC	Oil-uptake capacity
